# *Fto*-Deficiency Affects the Gene and MicroRNA Expression Involved in Brown Adipogenesis and Browning of White Adipose Tissue in Mice

**DOI:** 10.3390/ijms17111851

**Published:** 2016-11-07

**Authors:** Justiina Ronkainen, Eleonora Mondini, Francesca Cinti, Saverio Cinti, Sylvain Sebért, Markku J. Savolainen, Tuire Salonurmi

**Affiliations:** 1 Biocenter Oulu, University of Oulu, FI-90220 Oulu, Finland; sylvain.sebert@oulu.fi (S.S.); markku.savolainen@oulu.fi (M.J.S.); tuire.salonurmi@oulu.fi (T.S.); 2 Faculty of Medicine, Department of Internal Medicine, University of Oulu, FI-90220 Oulu, Finland; 3 Medical Research Center Oulu, Oulu University Hospital and University of Oulu, FI-90220 Oulu, Finland; 4 Department of Experimental and Clinical Medicine, Marche Polytechnic University, IT-60126 Ancona, Italy; ele_mondini@yahoo.it (E.M.); cinti_francesca@hotmail.com (F.C.); cinti@univpm.it (S.C.); 5 Center for Life-Course Health Research, University of Oulu, FI-90220 Oulu, Finland

**Keywords:** FTO, brown adipose tissue (BAT), white adipose tissue (WAT) browning, gene expression, microRNA expression, high-fat diet

## Abstract

Genetic variants in the fat mass- and obesity-associated gene *Fto* are linked to the onset of obesity in humans. The causal role of the FTO protein in obesity is supported by evidence obtained from transgenic mice; however, the underlying molecular pathways pertaining to the role of FTO in obesity have yet to be established. In this study, we investigate the *Fto* gene in mouse brown adipose tissue and in the browning process of white adipose tissue. We analyze distinct structural and molecular factors in brown and white fat depots of *Fto*-deficient mice under normal and obesogenic conditions. We report significant alterations in the morphology of adipose tissue depots and the expression of mRNA and microRNA related to brown adipogenesis and metabolism in *Fto*-deficient mice. Furthermore, we show that high-fat feeding does not attenuate the browning process of *Fto*-deficient white adipose tissue as observed in wild-type tissue, suggesting a triggering effect of the FTO pathways by the dietary environment.

## 1. Introduction

Common genetic variants in the fat mass- and obesity-associated gene *Fto* are the most prevalent variants linked to higher body mass index (BMI). The genetic association is robust and consistently described in several age and ethnic groups [1,2,3,4,5]. The “obesogenic environment” in the world, with increased availability of cheap, calorie rich food has a major role in the current obesity epidemic, but individuals respond to it differently. While genetic factors are estimated to account for 40%–70% of BMI variation, only a fraction of it is explained by presently known genetic variants [6]. This may in part be due to the limited amount of genetic variants yet identified, but there is also growing evidence of a gene-environment interaction, which needs to be fully established when the genetics of obesity is under investigation (reviewed in [7]). For example, individuals homozygous for the obesity-risk allele of *Fto* variant rs9939609 showed improved weight loss and metabolic markers after low-fat, but not low-carbohydrate, hypocaloric dietary intervention, while the improvement was evident on both diets in the group of the protective allele carriers [8]. Furthermore, a cohort-based study showed a different effect of genetic variants on the risk of obesity depending on the person’s year of birth. In that study, one risk allele increase led to a 0.16 increase in BMI among men born in 1930 compared with a 0.47 increase among those born in 1970 [9]. As the environment becomes more “obesogenic”, individuals with higher genetic predisposition for obesity gain more weight compared with lower genetic risk carriers. Thus, in addition to genetic variants affecting BMI, it has become important to assess the gene-environment interaction, not only to estimate the risk of obesity, but also to find suitable methods for solving the world-wide obesity epidemic.

Transgenic mouse models indicate that the FTO protein has an independent role in energy metabolism, with *Fto*-knockout (*Fto*-KO) mice having reduced body weight and the overexpression of *Fto* leading to an obese phenotype in mice [10,11,12,13,14]. *Fto* mRNA is ubiquitously expressed in such mouse tissues as brain, skeletal muscle, pancreas, liver, heart, spleen, kidney, lung, as well as white and brown adipose tissues (WAT and BAT, respectively) [15,16]. Recently, we reported that the invalidation of the *Fto* gene in mice could play a role in the metabolism of WAT and distinctively observed that the alteration is specific to the dietary environment [17]. Critically, *Fto*-KO mice do not develop the diet-induced obese phenotype and the white adipocytes do not become hypertrophic in response to a high-fat diet (HFD) despite unchanged eating behavior, respiratory exchange ratio, and activity in comparison to wild-type (WT) mice. This led to the question of whether *Fto* participates in WAT development and/or metabolism, and interestingly, we found altered in vivo expression of genes related to adipogenesis in *Fto*-KO epididymal WAT [17].

Recent evidence suggests that the FTO protein could also affect the biology of brown adipose tissue (BAT) and the browning process of WAT [18]. In the browning process, white adipocytes begin to resemble brown adipocytes and utilize energy instead of conserving it. These adipocytes are referred to as beige or brite (brown-in-white) adipocytes, sharing precursor cells mainly with white adipocytes. Despite originating from distinct precursor cells, brown and white adipogenesis are conservatively regulated by CCAAT/enhancer binding protein (C/EBP) and peroxisome proliferator activated receptor (PPAR) families of transcription factors (reviewed in [19]). C/EBPβ cooperates with the positive regulatory domain containing 16 (PRDM16) to induce brown fat determination and differentiation, which subsequently leads to activation of thermogenic genes such as adrenergic receptor β3 (*Adrb3*), peroxisome proliferator-activated receptor γ coactivator 1α (*Pgc1a*), and mitochondrial uncoupling protein 1 (*Ucp1*) [20,21]. These markers are also characteristic to beige adipocytes [22]. FTO is found to remove methyl groups from the N6-position of adenosine in single-stranded RNA, a modification also described for microRNA (miRNA) binding sites, and a recent finding indicates FTO participates in the demethylation of miRNAs [23,24,25]. miRNAs are RNA molecules of 21 to 23 nucleotides in length that participate in post-transcriptional regulation of gene expression and are involved in important cellular pathways including differentiation, division, and apoptosis [26]. Several individual miRNAs have been shown to participate in the fine-tuning of genes important in both white and brown adipogenesis (reviewed in [27,28,29]). These include the miR-130 family members that repress brown and white adipogenesis via direct inhibition of *Pparg* [30] and miR-378 that activates *Cebpa* and *Cebpb* expression during adipogenesis and enhances brown fat expansion [31,32]. In the WAT, miR-155 regulates the browning process by reducing the expression of *Cebpb*, *Pgc1a*, and *Prdm16* [33].

In the current study, we aim to learn new effects of FTO in BAT as well as in the browning process of WAT in vivo. We and others have shown that FTO affects genes regulating adipogenesis [17,18,34] and now we also intend to find out how distinct miRNAs might be linked to this effect. As FTO is shown to regulate the level of miRNAs [25], we also investigated the miRNA expression in BAT and WAT depots in response to *Fto*-KO and HFD. BAT and subcutaneous WAT of wild-type and *Fto*-KO mice were studied and the mice were exposed to HFD to investigate the interplay of *Fto* with an obesogenic environment.

## 2. Results

### 2.1. Phenotype, Adipose Tissue Morphology, and Uncoupling Protein 1 (UCP1) Immunohistochemistry

**Figure 1:**
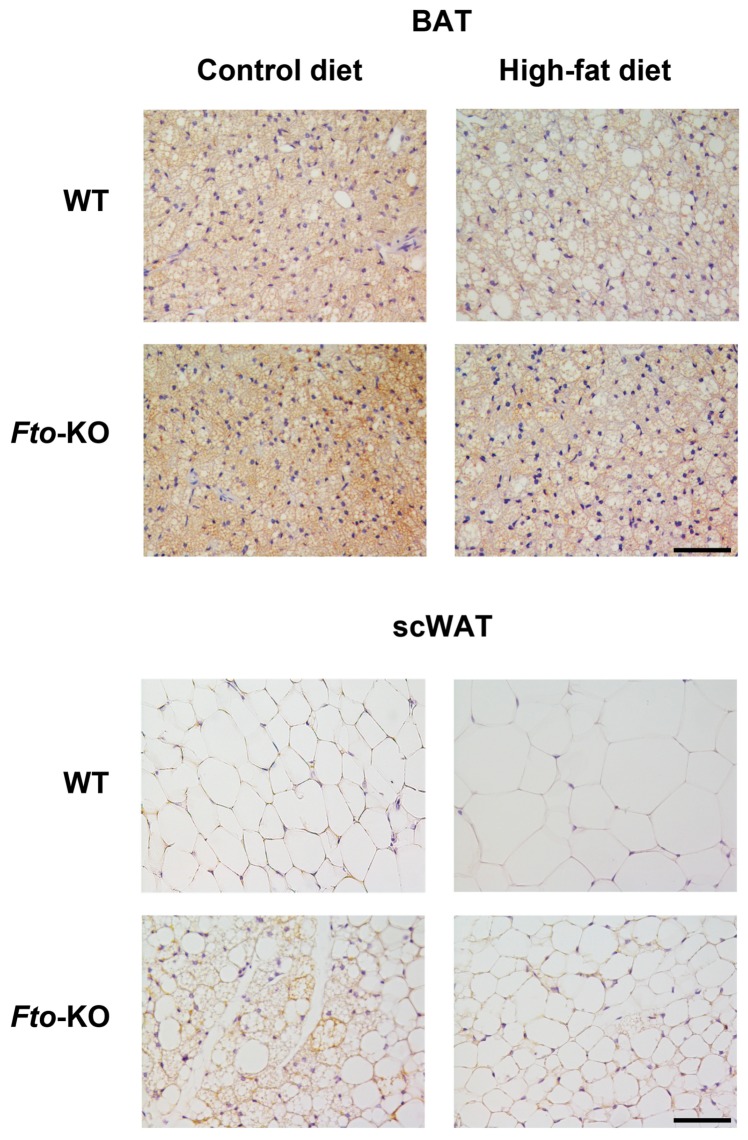
Uncoupling protein 1 (UCP1) immunohistochemistry of interscapular brown adipose tissue (BAT) and subcutaneous white adipose tissue (scWAT). WT, wild-type mice; *Fto*-KO, *Fto*-knockout mice. Scale bars are 60 µm and images are a representative of two to three different mice in each group.

**Figure 2:**
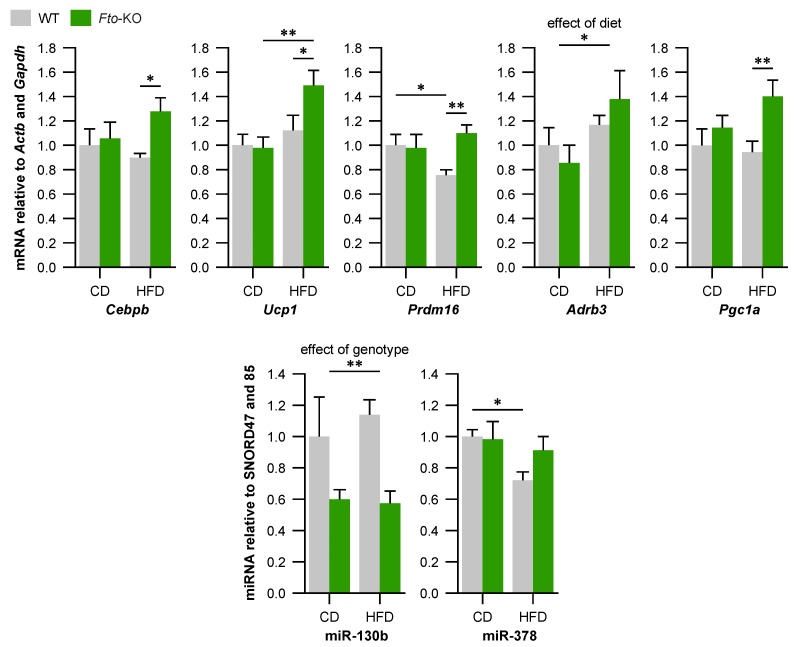
Relative expression of mRNAs and miRNAs related to BAT adipogenesis and metabolism in interscapular BAT. The amount of mRNA was normalized using *Actb* and *Gapdh* as reference genes and the amount of miRNA was normalized using SNORD47 and SNORD85 as internal controls. WT, wild-type mice; *Fto*-KO, *Fto*-knockout mice; CD, control diet; HFD, high-fat diet. Results are shown as mean ± SEM (*n* = 5–9 per group in mRNA, *n* = 4 per group in miRNA). Two-way ANOVA followed by simple main effects analysis with Bonferroni correction if there was significant interaction effect or moderation effect, * *p* < 0.05, ** *p* < 0.01.

The phenotype of *Fto*-KO mice was described previously in more detail [17]. Briefly, the weight of *Fto*-KO mice was lower than that of WT mice when the mice were fed HFD (WT 45.4 g (1.07) vs. KO 32.3 g (1.64), *p* < 0.001), indicating a resistance to diet-induced obesity, while the energy intake was unchanged between the two groups. Interestingly, metabolic and physical parameters such as heat production, respiratory exchange rate, and activity were unaltered between the genotypes [17]. UCP1 immunohistochemistry of BAT and subcutaneous WAT (scWAT) specimens are shown in Figure 1. The UCP1 protein was most highly expressed in the BAT of *Fto*-KO mice, which was also seen in *Ucp1* mRNA expression when the mice were fed HFD (Figure 2). Adipocytes in the scWAT of WT mice were unilocular and UCP1-negative, whereas *Fto*-KO scWAT contained multilocular, UCP1-positive cells. When fed HFD, scWAT adipocytes from WT mice were unilocular, hypertrophic, and UCP1-negative, while *Fto*-KO adipocytes were small unilocular with occasional multilocular and UCP1-positive cells (Figure 1). Phenotypic differences were more subtle when mice were fed the control diet (CD) in comparison to HFD.

### 2.2. Expression of Brown Adipocyte Markers Is Altered in Brown Adipose Tissue (BAT) of Fto-Knockout (Fto-KO) Mice

As shown in Figure 2, the expression of *Cebpb* mRNA was increased by 1.3-fold in *Fto*-KO BAT on HFD. This was not observed in WT mice. Furthermore, *Prdm16* expression was at the same level in *Fto*-KO BAT on both diets, while HFD-fed WT BAT showed a reduction of *Prdm16* expression by 1.3-fold. While there was no difference in the relative expression of *Ucp1* between WT and *Fto*-KO BAT on CD, its expression was increased by 1.5-fold in the BAT of *Fto*-KO mice fed HFD but not in WT mice. *Adrb3* expression was increased in BAT of HFD-fed mice by 1.2-fold and 1.4-fold in the WT and *Fto*-KO, respectively. Expression of *Pgc1a* was increased by 1.4-fold in *Fto*-KO mice fed HFD but this effect was not observed in WT mice. Expression of *Fto* was absent in *Fto*-KO BAT and there was no difference in *Fto* expression between CD- and HFD-fed WT mice. Expression of miR-130b, a repressor of *Pparg* [30], was decreased 1.7-fold in *Fto*-KO mice compared with WT irrespective of the diet (Figure 2). miR-378 has been shown to activate *Cebpa* and *Pparg* [31,35]. In concert with this, the expression of miR-378 was at the same level in *Fto*-KO BAT of mice fed either diet while in the WT BAT, miR-378 was decreased by 1.4-fold after HFD. Expression of several miRNAs related to BAT metabolism such as miR-26a, miR-27a, miR-93, miR-193b, miR-365a, and miR-445 were altered in response to the diet in both genotypes while no effect of genotype was observed (Figure S1).

### 2.3. Beige Adipocyte Markers Are Expressed Higher in Fto-KO Subcutaneous White Adipose Tissue (WAT)

**Figure 3:**
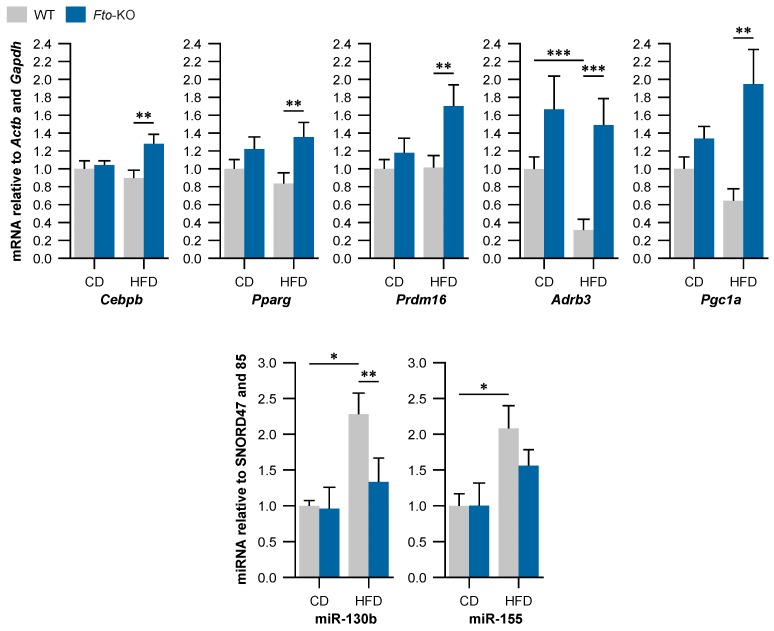
Relative expression of mRNAs and miRNAs related to WAT browning in subcutaneous WAT. The amount of mRNA was normalized using *Actb* and *Gapdh* as reference genes and the amount of miRNA was normalized using SNORD47 and SNORD85 as internal controls. WT, wild-type mice; *Fto*-KO, *Fto*-knockout mice; CD, control diet; HFD, high-fat diet. Results are shown as mean ± SEM (*n* = 5–9 per group in mRNA, *n* = 4 per group in miRNA). Two-way ANOVA followed by simple main effects analysis with Bonferroni correction if there was significant interaction effect or moderation effect, * *p* < 0.05, ** *p* < 0.01, *** *p* < 0.001.

In the scWAT of *Fto*-KO mice, the expression of *Cebpb* was increased by 1.4-fold and *Pparg* by 1.6-fold relative to WT when the mice were fed HFD (Figure 3). *Prdm16* expression was increased by 1.7-fold in the scWAT of *Fto*-KO mice fed HFD while an increase was not observed in WT mice. Expression of *Ardb3* was at the same level in scWAT of *Fto*-KO mice fed either diet, while it was decreased by 3.1-fold in scWAT of WT mice fed HFD. In fact, *Adrb3* was expressed 4.7-fold higher in *Fto*-KO fed HFD compared with WT fed HFD. Expression of *Pgc1a* was increased by 3-fold in scWAT of *Fto*-KO mice fed HFD when compared with WT mice fed HFD. Similar to BAT, *Fto* expression was absent in *Fto*-KO scWAT and no difference was observed in *Fto* expression between CD- and HFD-fed WT mice. Furthermore, HFD induced a 2.3-fold increase in miR-130b, a repressor of *Pparg* [30], in the scWAT of WT mice but not in *Fto*-KO mice. A similar pattern was observed in the expression of miR-155 (Figure 3).

## 3. Discussion

We reported recently that the epididymal WAT of *Fto*-KO mice is distinct from WT which affects its adaptation to high-fat feeding [17]. These mice do not develop diet-induced obesity or adipocyte hypertrophy on HFD, while the eating behavior, respiratory exchange ratio, heat generation, and activity are similar to WT [17]. The current study focuses on the BAT and scWAT of the same animals to further untangle the effects of FTO upon BAT metabolism and browning of WAT. ScWAT was specifically chosen because among all WAT depots, as it has been shown to be the most susceptible to browning (reviewed in [36]). We found that brown and beige adipocyte markers were not attenuated in *Fto*-KO mice on HFD, a change we observed in WT. Furthermore, we found differently expressed miRNAs between *Fto*-KO and WT which may contribute to this adaptation to the dietary environment.

Our results show that *Fto*-KO brown adipocytes were smaller with higher expression of BAT markers on HFD when compared with WT. Furthermore, HFD induced an accumulation of lipids into BAT of WT mice, which was not observed in *Fto*-KO mice. In line with Tews et al. [18], our results show that *Fto*-KO scWAT was more prone to browning on HFD than WT. This suggested a role for FTO in the plasticity of the adipose tissue. The present study shows that the adipocytes in *Fto*-KO scWAT were more often multilocular and UCP1-postive compared with WT and *Fto*-KO adipocytes that did not become hypertrophic on HFD. This phenomenon was also seen in the epididymal WAT from our previously published observations [17].

In this study, the fat mass was not analyzed separately from lean mass. However, previous studies with *Fto* mouse models indicate that the fat mass of *Fto*-deficient mice is either reduced [10,11,12] or unaltered [13] compared with WT. Furthermore, the International Mouse Phenotyping Consortium database (www.mousephenotype.org) show unaltered fat mass for the mouse model similar to one presented here (Fto^tm1a(EUCOMM)Wtsi^) [37]. Thus, in the light of previous reports, we may postulate that the fat mass of current *Fto*-KO mice is not increased. Previous studies report *Fto*-KO mice to manifest increased plasma and WAT adiponectin, improved glucose tolerance, and reduced adipocyte size [10,12,17]. Mild improvement in insulin sensitivity is also reported, however it is speculated to be likely a consequence of increased circulating adiponectin [10]. Since *Fto*-KO mice are not completely devoid of fat, FTO does not seem solely necessary for adipocyte differentiation. However, the resistance to diet-induced obesity observed in the current *Fto*-KO mice indicate an improved ability of adipose tissue to respond to environmental changes such as HFD. This is important with regards to human genetics of obesity, since environmental factors have been shown to alter the effects of genetic variants in individual’s risk of obesity and assessment of these factors may help in choosing the most suitable interventions in the prevention and management of obesity (reviewed in [7]).

Morphological alterations observed here were further supported by the changes in gene and miRNA expression related to BAT adipogenesis and function as well as the browning of WAT. Expression of BAT markers were altered together with the expression of miRNAs reported to participate in the brown and white adipogenesis as well as in the browning process. *Ucp1* mRNA expression in BAT was unaltered between WT and *Fto*-KO mice on CD, but *Fto*-KO mice showed an increase in *Ucp1* expression on HFD. This is in line with Fischer et al. [10] as they report unaltered BAT morphology and *Ucp1* expression of *Fto*-KO mice; however, in the study by Tews et al. [18] *Fto*-KO mice were reported to demonstrate increased *Ucp1* expression even on CD and the effect was more pronounced in that study. This phenotypic difference may be due to fact that the mice in the current and in the Fischer et al. [10] studies were older (22 and 20 weeks, respectively) than in the study by Tews et al. (11 weeks) [18]. Furthermore, due to the experimental conditions of the current study, UCP1 immunohistochemical analysis was conducted on 15-week-old mice and indeed, the UCP1 protein expression was increased in *Fto*-KO BAT even on CD as manifested by immunohistochemistry. Age dependence is also apparent in human studies, where association of *FTO* variants with BMI is more strongly observed in children but tends to be lost in older age [5,38]. Thus, these effects may be more pronounced in younger animals in which the body weight has not yet diverged, a notion which warrants further investigation in the near future. Previous reports concerning the effects of HFD on *Ucp1* expression are controversial as there are studies reporting *Ucp1* to be increased, decreased, and unchanged due to HFD. This discrepancy was speculated to result from different experimental factors such as very low fat levels of CD or the different fatty acid composition between diets rather than the actual effect of HFD, which warrants further clarification elsewhere (reviewed in [39]). In the current study, the CD and HFD contained the same amount of protein, while the relative fat content was increased at the expense of carbohydrates in HFD. As protein or carbohydrate content is not reported to affect *Ucp1* expression [39] and the fat source was the same in both diets, we can safely assume that the increase in *Ucp1* expression observed in *Fto*-KO mice is indeed due to HFD. *Ucp1*-deficient mice become obese in thermoneutrality, indicating anti-obesogenic effects for UCP1 [40].

The present results strongly support the notion that FTO participates in regulation of miRNA expression and through that, affects the adipose tissue plasticity [25]. Expression of miR-130 is increased in adipocyte hypertrophy and fat inflammation [41] and interestingly, miR-130 of the current *Fto*-KO BAT was significantly decreased in comparison with WT, indicating a role for FTO in the regulation of miR-130 and the pathophysiology of obesity. In addition, miR-378 was reported to activate C/EBPs during adipogenesis and enhance BAT expansion [31,35]. C/EBPβ together with PRDM16 acts during the early phase of brown adipocyte differentiation and affects the expression of BAT marker genes [21] which was also observed in our study. Interestingly, the *Cebpb* expression was increased due to HFD in epididymal WAT of both WT and *Fto*-KO mice [17,42], but was unaltered in BAT and scWAT of the current WT mice. Thus, the HFD-induced increase in *Cebpb* expression may be adipose tissue-specific, which is an interesting finding worthy of further investigation. *Fto* may have a role in this tissue-specificity, since *Cebpb* expression was increased in *Fto*-KO BAT and scWAT when mice were fed HFD.

WAT adipocytes of the *Fto*-KO mice were more often multilocular and UCP1 positive in comparison with WT. This is common for beige adipocytes i.e., white adipocytes manifesting brown characteristics such as increased energy expenditure and decreased fat storage, as well as increased expression of BAT marker genes [22]. Thus, the absence of FTO possibly improves the adaptation of WAT into an altered dietary environment via enhanced browning. PPARγ and C/EBPβ are important transcription factors in both brown and white adipogenesis and are inhibited by miR-130 and miR-155, respectively [30,33]. In addition, miR-130 was reported to be increased in WAT of mice due to HFD [41], which was also observed in our WT mice but not in the *Fto*-KO mice. In the current study, the expression of these anti-adipogenic miRNAs was increased in WT mice on HFD with the concurrent decrease in adipogenic *Pparg* and *Cebpb* expression. These transcription factors subsequently regulate downstream BAT marker genes such as *Prdm16*, *Adrb3*, and *Pgc1a* [19].

The most striking effect of *Fto*-KO on gene expression was found in mice exposed to high-fat feeding. This implicates an altered capability of *Fto*-KO BAT and scWAT to adapt to changes in the dietary environment. This was also seen in the epididymal WAT and genes related to white adipogenesis of these mice [17]. Interestingly, in the current study many of the miRNAs related to BAT metabolism and function were altered due to HFD without a difference between WT and *Fto*-KO. This indicates that there is also an FTO-independent role for miRNA expression in the adaptation of BAT to high-fat feeding and an obesogenic environment. Indeed, several miRNAs have been shown to be regulated in response to HFD and obesity and further studies in this field are definitely required [43,44,45].

FTO removes methyl groups from *N*6-methyladenosine (m6A) in RNA, and while the modification has been known to exist for several decades, its physiological role is only currently being resolved [23,24,46]. In addition to FTO, one other m6A demethylase, AlkB-homolog 5 (ALKBH5), has been identified [47]. ALKBH5, as well as FTO to some extent, co-localizes with nuclear speckles, which are nuclear compartments containing mRNA processing factors, indicating a role for FTO in the splicing and/or export of RNA from the nucleus to the cytoplasm [23,47]. *Fto*-knockdown has been shown to affect the level of certain miRNAs and 67% of the mRNA 3′ untranslated regions (3′UTRs) with m6A, contained also one or more predicted miRNA binding sites, supporting a role for m6A and FTO in the miRNA biology [24,25]. Furthermore, a decrease in m6A results in downregulation of mature miRNA expression and accumulation of unprocessed pre-miRNA in several cell lines, indicating that m6A is necessary for miRNA processing [48]. Thus, the regulation of RNA methylation is clearly important and is an intriguing new field of epigenetics that demands further investigation.

In conclusion, our study supports a role for FTO in BAT metabolism and the browning process of WAT. Our present data indicate that this adaptation could be primarily regulated by changes in miRNA expression. This is supported by the fact that FTO is a demethylase of m6A and a recently proposed link between miRNA signaling and m6A methylation [23,24,25]. As the knowledge of the actual physiological role of m6A methylation and miRNA signaling will expand, we might expect that our unique finding will contribute to the understanding of the FTO’s mechanism of action in the near future.

## 4. Materials and Methods

### 4.1. Generation of Fto-KO Mice and Dietary Treatment

The *Fto*-KO embryonic stem cells were purchased from EUCOMM (Available online: www.eucomm.org, clone EPD0103_5_G10) and the generation and genotyping protocol of the mouse line has been previously described [17]. The KO-construct contained a polyadenylation signal and caused termination of *Fto* gene transcription inside the second intron. Mice were housed in groups in the Laboratory Animal Center, University of Oulu, at 21 ± 2 °C on a 12/12-h dark-light cycle and had ad libitum access to water and either the control or high-fat diet. CD contained 10 kJ% fat, 20 kJ% protein, and 70 kJ% carbohydrate (total energy 16.1 kJ·g^−1^), while HFD had 45 kJ% fat, 20 kJ% protein, and 35 kJ% carbohydrate (total energy 19.8 kJ·g^−1^) (D12450B and D12451, Research Diets, New Brunswick, NJ, USA). Six-week-old male mice were randomly allocated into four groups: WT on CD (*n* = 9), *Fto*-KO on CD (*n* = 5), WT on HFD (*n* = 9), and *Fto*-KO on HFD (*n* = 8), and were sacrificed at the age of 22 weeks by carbon dioxide inhalation. Interscapular BAT and scWAT samples were collected in liquid nitrogen and stored at −70 °C. Animal experiments were approved by the University of Oulu Animal Ethics Committee and the Southern Finland Regional State Administrative Agency (license numbers ESLH-2008-06715/Ym-23 and ESAVI/4464/04.10.03/2011). The animal care and procedures were carried out in accordance with the national Finnish legislation, the European Convention for the protection of vertebrate animals used for experimental and other scientific purposes (ETS 123), and EU Directive 2010/63/EU.

### 4.2. Immunohistochemistry

After dissection, adipose tissue depots were fixed by immersion in 4% paraformaldehyde in 0.1 M phosphate buffer pH 7.4 overnight at 4 °C, and then dehydrated, cleared, and paraffin embedded. Serial paraffin sections of 3 µm were obtained, one for hematoxylin and eosin staining to assess morphology, and the others for immunohistochemical processing. Morphology and UCP1 protein expression in the BAT and scWAT were studied from two to three mice per group, two mice in scWAT of the WT HFD group, and three mice in the remaining. Tissue sections were imaged with a Nikon Eclipse E800 light microscope using a 40× objective and digital images were captured with a Nikon DXM 1200 camera. For immunohistochemistry, 3 μm dewaxed serial sections were incubated with anti-UCP1 (1:200 for BAT and 1:1000 for scWAT, rabbit polyclonal anti-UCP1, Abcam, Cambridge, UK) primary antibody according to the Avidin Biotin Complex method. To inactivate endogenous peroxidase, 0.3% hydrogen peroxide was used followed by normal goat serum to reduce nonspecific staining. The sections were incubated overnight at 4 °C with primary antibodies. The biotinylated HRP-conjugated secondary antibody was goat anti-rabbit IgG (Vector Laboratories; Burlingame, CA, USA). Histochemical reactions were performed using Vector’s Vectastain ABC Kit (Burlingame, CA, USA) and Sigma Fast 3,3′-diaminobenzidine as the substrate (Sigma, St. Louis, MO, USA). Sections were counterstained with hematoxylin.

### 4.3. Isolation of Total RNA and miRNA-Enriched Fraction and cDNA Synthesis

Total RNA was isolated from 50 to 100 mg of BAT and scWAT. Tissues were homogenized with QIAzol Lysis Reagent and TissueLyser LT according to the manufacturer’s instructions (Qiagen, Hilden, Germany). From BAT, miRNA-included total RNA was isolated with the miRNeasy Mini Kit, whereas from scWAT, total RNA and miRNA-enriched fraction were isolated separately with the RNeasy Mini Kit and the RNeasy MinElute Cleanup Kit (Qiagen, Hilden, Germany). The concentration and quality of the total RNA were analyzed with a NanoDrop ND-1000 UV-Vis Spectrophotometer (NanoDrop Technologies, Wilmington, DE, USA) and the concentration of miRNA-enriched fraction with the RediPlate 96 RiboGreen RNA Quantitation Kit (Molecular Probes, Eugene, OR, USA). Genomic DNA was removed from the total RNA with DNAase treatment and cDNA was synthetized from 500 ng of total RNA using the RevertAid™ First Strand cDNA Synthesis Kit (Fermentas, Sankt Leon-Rot, Germany). From scWAT, miRNA cDNA synthesis was conducted from 100 ng of miRNA-enriched fraction while from BAT, miRNA cDNA was synthesized from 500 ng of total RNA with the qScript microRNA cDNA Synthesis Kit (Quanta BioSciences, Gaithersburg, MD, USA).

### 4.4. Quantitative Real-Time Reverse-Transcriptase Polymerase Chain Reaction (RT-qPCR)

Relative expression of genes and miRNAs related to BAT adipogenesis and function as well as the browning process of scWAT was studied. For gene expression, β-actin (*Actb*) and glyceraldehyde-3-phosphate dehydrogenase (*Gapdh*) were used as reference genes and all the primers used in the gene expression analysis were obtained from Sigma-Aldrich (St. Louis, MO, USA). For miRNA expression, non-coding RNAs SNORD47 and SNORD85 were used as internal references and the primers were obtained from Quanta BioSciences (Gaithersburg, MD, USA).

The gene expression RT-qPCR reactions were performed with iQ SYBR Green Supermix (Bio-Rad Laboratories, Hercules, CA, USA) according to the manufacturer’s instructions. The PCR protocol was: 3 min 95 °C; 40 cycles of 10 s 95 °C, 10 s annealing temperature, 10 s 72 °C; 2 min 72 °C. Melting curve analysis was included at the end of every run: 55 to 95 °C at 0.5 °C intervals, 10 s at each temperature. miRNA expression RT-qPCR reactions were performed with PerfeCTa SYBR Green SuperMix (Quanta BioSciences, Gaithersburg, MD, USA) according to the manufacturer’s instructions. The miRNA PCR protocol was: 2 min 95 °C; 40 cycles of 5 s 95 °C, 30 s annealing temperature; melt curve: 65 to 95 °C at 0.5 °C intervals, 5 s at each temperature. Annealing temperatures as well as primer information are shown in Tables S1 and S2. Gene expression *C*_t_ values were collected using an iCycler thermal cycler and iQ5 Real-Time Detection System and miRNA expression *C*_t_ values with a CFX96 Real-Time PCR Detection System (Bio-Rad Laboratories, Hercules, CA, USA). Relative expression of each gene and miRNA was calculated using the 2^−ΔΔ*C*t^ method [49]. Each sample was analyzed in duplicates and an interrun calibrator and no-template control were used in the plates.

### 4.5. Statistics

For the relative expression of genes and miRNAs, two-way ANOVA and simple main effects analysis with Bonferroni correction were conducted if there was a significant interaction effect or possible moderation effects. The Shapiro-Wilk test and Levene’s test were used to evaluate the distributions of the variables and equalities of variance, respectively. Logarithmic transformation was applied for skewed distribution and/or unequal variance. *p* values were considered statistically significant when lower than 0.05 and the results were indicated as mean and SEM. Statistical analyses were conducted with IBM SPSS Statistics version 20.0 (IBM, Armonk, NY, USA).

## Supplementary Materials

Supplementary materials can be found at www.mdpi.com/1422-0067/17/11/1851/s1.

## Abbreviations

*Actb*	β-actin gene
*Adrb3*	adrenergic receptor β3 gene
ALKBH5	AlkB-homolog 5
BAT	brown adipose tissue
BMI	body mass index
brite	brown-in-white
*Cebpb*	CCAAT/enhancer binding protein β gene
*Fto*	fat mass- and obesity-associated gene
*Fto*-KO	*Fto*-knockout
*Gapdh*	glyceraldehyde-3-phosphate dehydrogenase gene
m6A	*N*6-methyladenosine
miRNA	microRNA
*Pgc1a*	peroxisome proliferator-activated receptor γ coactivator 1α gene
*Pparg*	peroxisome proliferator activated receptor γ gene
*Prdm16*	positive regulatory domain containing 16 gene
scWAT	subcutaneous WAT
*Ucp1*	mitochondrial uncoupling protein 1 gene
WAT	white adipose tissue
