# Supplementary material for: Fto-Deficiency Affects the Gene and MicroRNA Expression Involved in Brown Adipogenesis and Browning of White Adipose Tissue in Mice
Source: Int J Mol Sci. 2016 Nov 7;17(11):1851. doi: 10.3390/ijms17111851 (PMC5133851; doi:10.3390/ijms17111851)
Supplement: Supplementary file 1 [file ijms-17-01851-s001.pdf]

Supplementary Materials: *Fto*-Deficiency Affects the Gene and MicroRNA Expression Involved in Brown Adipogenesis and Browning of White Adipose Tissue in Mice

Justiina Ronkainen, Eleonora Mondini, Francesca Cinti, Saverio Cinti, Sylvain Sebért, Markku J. Savolainen and Tuire Salonurmi

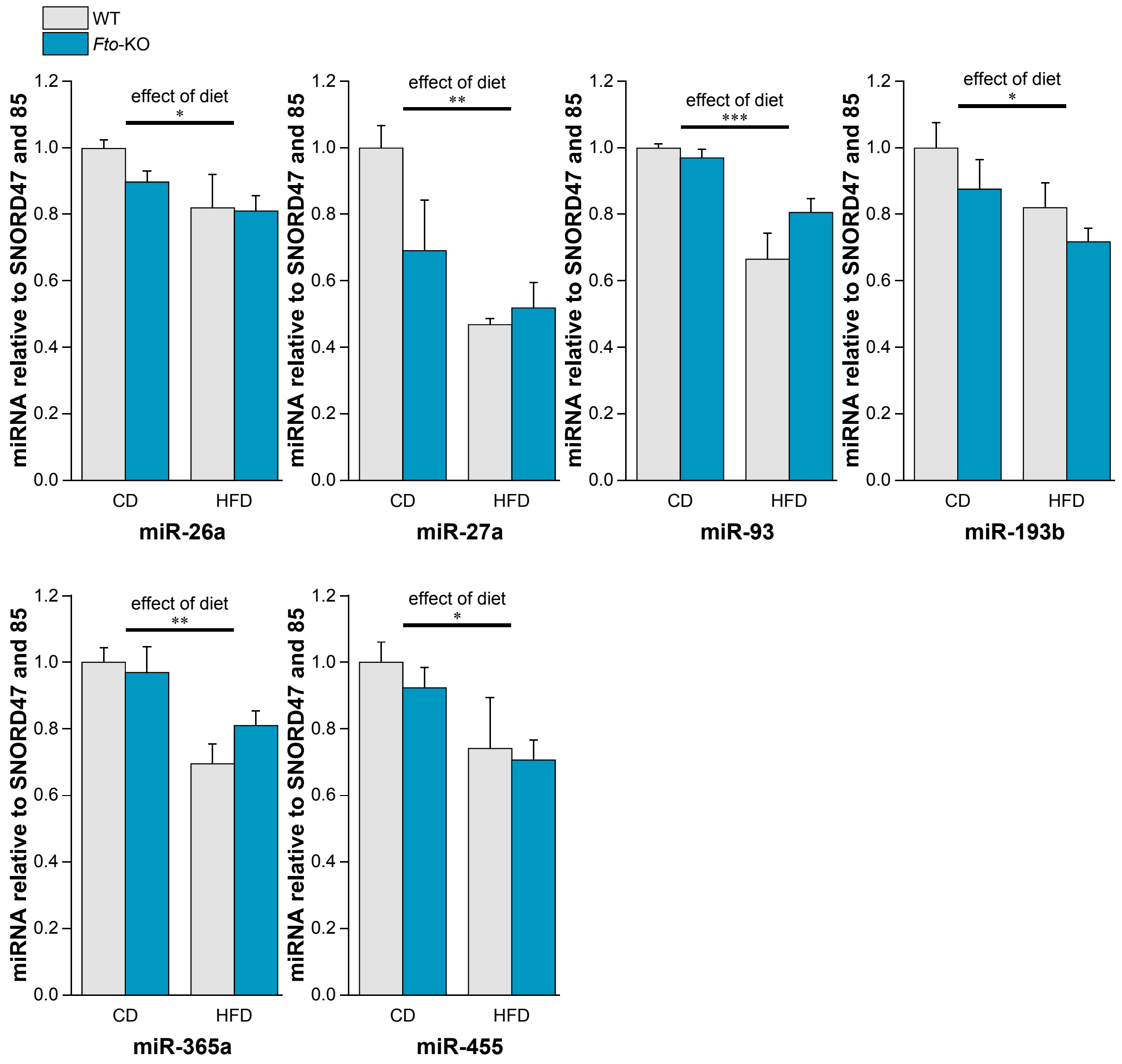

Figure S1. Relative expression of miRNAs related to brown adipose tissue (BAT) adipogenesis are downregulated due to the high-fat diet in BAT of wild-type (WT) and *Fto*-knockout (*Fto*-KO) mice. The amount of miRNA was normalized using SNORD47 and 85 as internal controls. CD, control diet; HFD, high-fat diet. Results are shown as mean ± SEM ($n = 4$ per group). Two-way ANOVA, * $p < 0.05$, ** $p < 0.01$, *** $p < 0.001$.

Table S1. Sequences and annealing temperatures (Ann °C) of primers used in the RT-qPCR studies.

Gene	Forward Primer 5′–3′	Reverse Primer 5′–3′	Ann °C
Actb	TGGATCGGTGGCTCCATCCTGG	CGCAGCTCAGTAACAGTCCGCCTA	61
Bmp4	AGCCGAGCCAACACTGTGAG	GAGCTCTGCCGAGGAGATCAC	62
Cebpa	ACGGCGGGAACGCAACAACA	GCTTGCGCAGGCGGTCATTG	62
Cebpb	CGCAACCTGGAGACGCAGCA	GGCTCGGGCAGCTGCTTGAA	61
Fto	GCGGAGGAACGAGAGCGGGA	GCTGCCGGCCTCTCGGAAAA	61
Gapdh	CCAATGTGTCCGTCGTGGATCT	GTTGAAGTCGCAGGAGACAACC	61
Glut4	GAGCTGGTGTGGTCAATACG	GTTCCAGCAGCAGCAGAG	60
Irx3	CACCCCGCCTTCTACCCCTA	TGGGTGAGGGTCATCTTGGTG	60
Pparg	ACTCCCTCATGGCCATTGAG	TGAGACATCCCCACAGCAAG	61
Rxra	CACCTGCCGAGACAACAAG	GTTCTCATTCCGGTCCTTGC	60
Ucp1	TGCCTCACTCAGGATTGG	GCTTGCATTCTGACCTTCAC	63

Table S2. Quanta BioSciences miRNA assay information and annealing temperatures (Ann °C) used in the miRNA RT-qPCR studies.

miRNA	Quanta BioSciences miRNA Assay (Ref#)	Ann °C
miR-26a	hsa-miR-26a-5p (68676137)	60
mir-27a	hsa-miR-27a * (68676138)	65
mir-93	HSMIR-0093 (69002641)	60
mir-106b	HSMIR-0106B (69002643)	60
mir-130b	MMIR-0130B * (69002636)	60
mir-133a	MMIR-0133A * (69002631)	60
mir-138	HSMIR-0138-5P (69002635)	60
mir-155	MMIR-0155 (69002637)	60
mir-193b	HSMIR-0193B-5P (69002642)	60
mir-196a	HSMIR-0196A-5P (69002638)	60
mir-200a	HSMIR-0200A-5P (69002639)	60
mir-365a	HSMIR-0365A-5P (69002640)	60
mir-378a	HSMIR-0378 * (69002632)	60
mir-448	MMIR-0448-5P (69002645)	60
mir-455	HSMIR-0455-5P (69002644)	60
SNORD47	MM-SNORD47 (69002634)	60
SNORD85	MM-SNORD85 (69002633)	60
